# Optogenetic stimulation of neurons in the anterior cingulate cortex induces changes in intravesical bladder pressure and the micturition reflex

**DOI:** 10.1038/s41598-024-56806-8

**Published:** 2024-03-16

**Authors:** Takanori Mochizuki, Satoshi Manita, Hiroshi Shimura, Satoru Kira, Norifumi Sawada, Haruhiko Bito, Kenji Sakimura, George J. Augustine, Takahiko Mitsui, Masayuki Takeda, Kazuo Kitamura

**Affiliations:** 1 Department of Urology, Faculty of Medicine, University of Yamanashi, Chuo, Yamanashi Japan; 2 Department of Neurophysiology, Faculty of Medicine, University of Yamanashi, Chuo, Yamanashi Japan; 3 Department of Neurochemistry, Graduate School of Medicine, The University of Tokyo, Tokyo, Japan; 4 Department of Cellular Neurobiology, Brain Research Institute, Niigata University, Niigata, Japan; 5 Temasek Life Sciences Laboratory, Singapore, Singapore

**Keywords:** Neurophysiology, Urology

## Abstract

Lower urinary tract (LUT) function is controlled by the central nervous system, including higher-order cognitive brain regions. The anterior cingulate cortex (ACC) is one of these regions, but the role of its activity in LUT function remains poorly understood. In the present study, we conducted optogenetic experiments to manipulate neural activity in mouse ACC while monitoring bladder pressure to elucidate how the activity of ACC regulates LUT function. Selective optogenetic stimulation of excitatory neurons in ACC induced a sharp increase in bladder pressure, whereas activation of inhibitory neurons in ACC prolonged the interval between bladder contractions. Pharmacological manipulation of ACC also altered bladder contractions, consistent with those observed in optogenetic experiments. Optogenetic mapping of the cortical area responsible for eliciting the increase in bladder pressure revealed that stimulation to ACC showed more potent effects than the neighboring motor cortical areas. These results suggest that ACC plays a crucial role in initiating the bladder pressure change and the micturition reflex. Thus, the balance between excitation and inhibition in ACC may regulate the reflex bidirectionally.

## Introduction

LUT function is known to be controlled by the periaqueductal gray matter (PAG) and the pontine micturition center (PMC)^1^ in the central nervous system. Patients with stroke and brain hemorrhage at the brainstem showed a high rate (49%) of urinary disturbance^2^. In contrast, lesions in the frontal cortex often resulted in urine storage disorders such as urinary frequency or urgency (68% of cases)^3^, indicating that the frontal cortex is believed to have suppressive effects on the micturition reflex. Human brain imaging studies using fMRI and PET have suggested that multiple brain regions such as PMC, PAG, ACC, thalamic nuclei, the insular cortex, and the prefrontal cortex are activated during micturition^4–6^. Moreover, optogenetic stimulation in mice revealed that a part of the primary motor area (M1) is also involved in micturition^7^. Among these areas, ACC is the most enigmatic. ACC was reported to be activated during the storage phase of micturition^8^, whereas activation of ACC in patients with overactive bladder^9^ and during the voiding phase^10^ was also reported. Therefore, the role of ACC in the micturition reflex, more specifically, whether ACC is activated during the storage or voiding phase remains highly controversial. In rats, electrical stimulation of ACC prolonged the intervals between bladder contractions, i.e., the micturition reflex was supposed to be suppressed by ACC activity^11^. However, the lack of stimulation specificity makes the interpretation of the results difficult.

In this study, we aim to elucidate the role of ACC in LUT functions by direct and selective manipulation of neural activity in ACC of mice during micturition. Excitatory or inhibitory neurons in ACC expressing channelrhodopsin-2 (ChR2) were photostimulated, while bladder pressure was monitored by using cystometry. Notably, we found that optogenetic stimulation of neurons in ACC of Thy1-ChR2 mice, which express ChR2 in layer 5 pyramidal neurons, induced a robust increase in bladder pressure time-locked to the onset of photostimulation. Photostimulation to mice locally expressing ChR2 in excitatory neurons of ACC induced similar effects. In contrast, photostimulation to mice expressing ChR2 in GABAergic interneurons in ACC prolonged interval between bladder contractions and the micturition. Taken together, the activity of ACC exerts bidirectional regulation over the bladder pressure change, whereby increased activity promotes and reduced activity suppresses it.

## Results

### Optogenetic stimulation of layer 5 pyramidal neurons in ACC of Thy1-ChR2 mice induces bladder pressure increase

**Figure 1:**
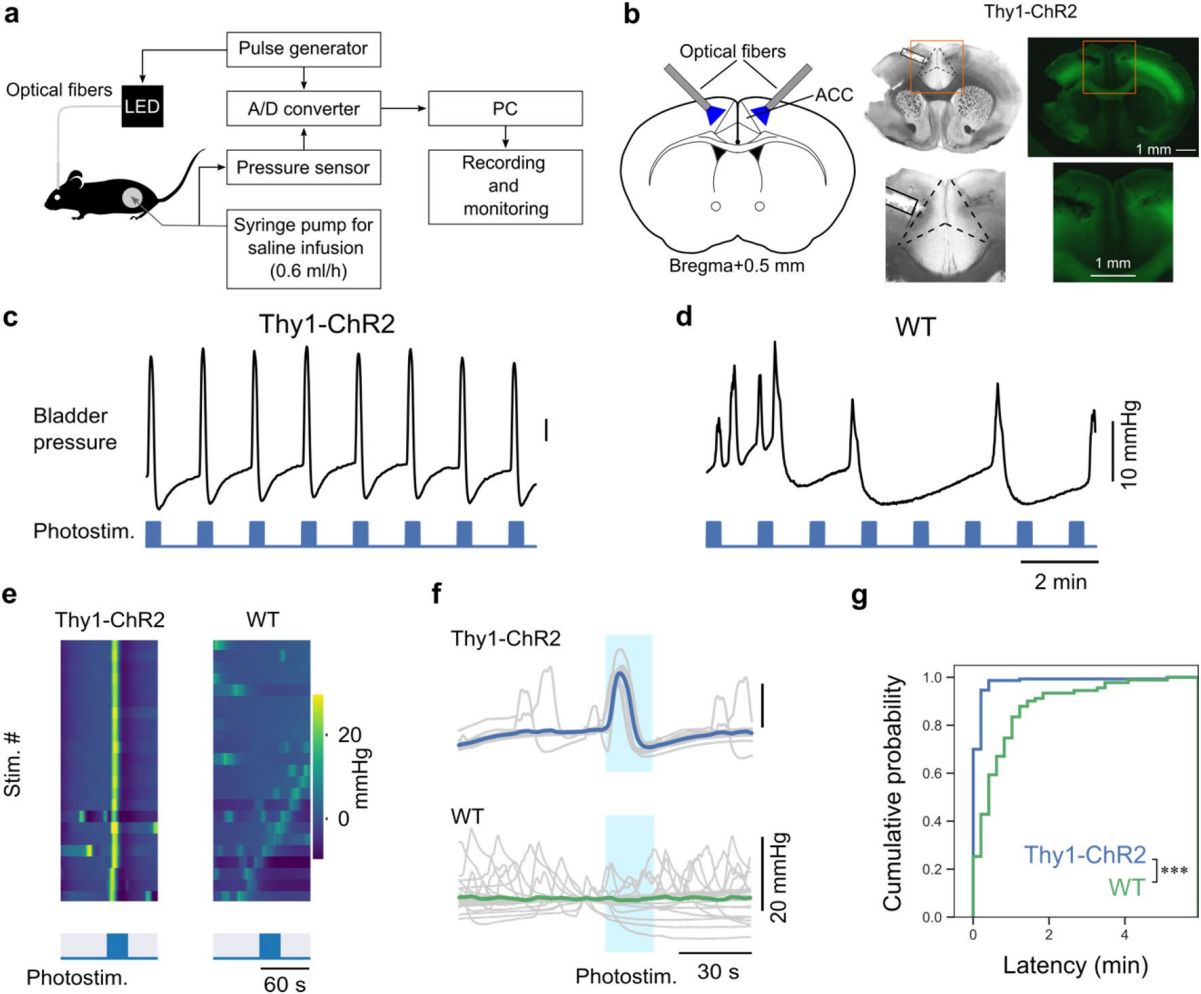
Optogenetic stimulation to ACC in Thy1-ChR2 mice reliably increases bladder pressure. (**a**) Schematic of the experiment. (**b**) Left, Optical fiber cannulas were implanted in bilateral ACC. Right, Fluorescence micrograph showing the expression of ChR2-EGFP in Thy1-ChR2 mice. (**c**, **d**) Representative examples of changes in bladder pressure during photostimulation of ACC in Thy1-ChR2 (**c**) and wild-type (**d**) mice. (**e**) Color plots showing bladder pressure changes for 1 min before and after the onset of photostimulation. (**f**) Data in e are shown overlaid as line plots. Colored lines indicate mean bladder pressures, and light gray lines indicate single-trial bladder pressures. The timing of the stimulus is indicated by light blue rectangles. (**g**) Cumulative plots of the latency from the onset of photostimulation to the nearest peak of the bladder pressure changes. Thy1-ChR2: 150 peaks from 4 mice, WT: 91 peaks from 8 mice. ***p < 0.001, Mann–Whitney U test.

We first examined whether manipulation of neural activity in ACC could affect bladder pressure. To directly confirm whether the activity of neurons in ACC triggers the bladder pressure change, we used an optogenetic approach (Fig. 1a). We first targeted excitatory projection neurons in ACC by using Thy1-ChR2 transgenic mice^12^. In this mouse line, most layer 5 pyramidal neurons express ChR2 throughout the cerebral cortex. When the bilateral ACC of Thy1-ChR2 mice was stimulated with a train of blue light pulses (Fig. 1b), bladder pressure was immediately and consistently elevated (Fig. 1c, and f). Stimulation at random light exposure timings also resulted in changes in bladder pressure (Supplementary Fig. S1). In contrast, photostimulation to ACC in WT mice failed to induce bladder pressure elevation (Fig. 1d–f). The latency of peak bladder pressure was precisely time-locked to the onset of photostimulation in Thy1-ChR2 mice but not in WT mice (median, Thy1-ChR2 mice: 7.2 s, 150 events, WT mice: 28.8 s, 176 events, Fig. 1g).

**Figure 2:**
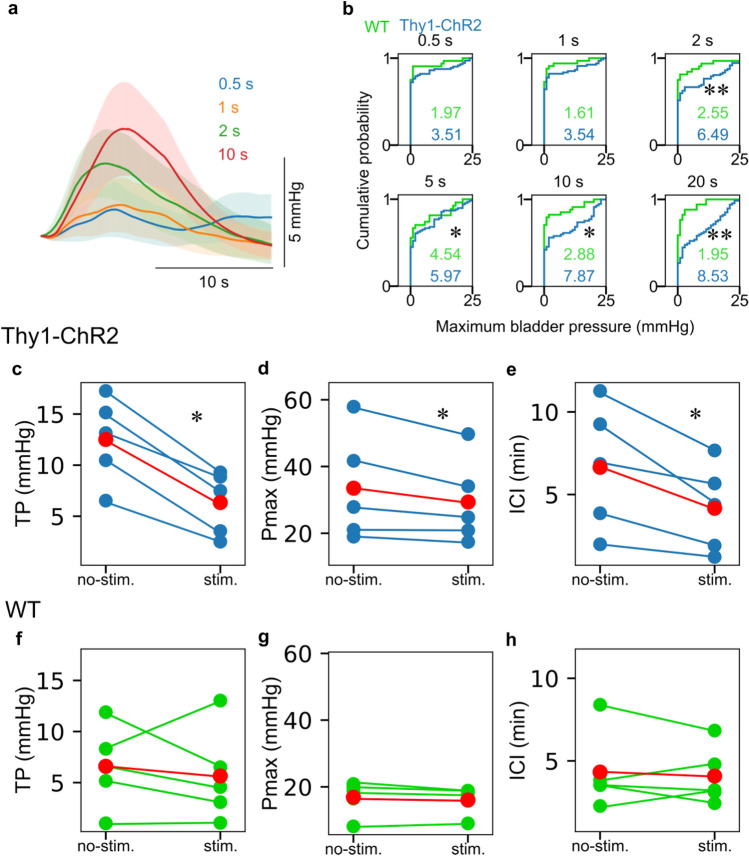
Effects of photostimulation to ACC in Thy1-ChR2 mouse. (**a**) Trial-averaged changes in the bladder pressure by photostimulation to bilateral ACC in Thy1-ChR2 mice. Thick lines and shadings indicate mean and 95% confidence interval, respectively. Colors indicate the duration of photostimulation. (**b**) Cumulative probability distributions of the maximum bladder pressure (Pmax) induced by photostimulation in Thy1-ChR2 and WT mice. Photostimulation over 2 s induced significantly larger Pmax in Thy1-ChR2 mice than in WT. The numbers in each graph represent the mean value of the maximum bladder pressure for each condition. n = 5 mice. *p < 0.05, **p < 0.01, Student’s t-test. (**c**–**e**) Effects of photostimulation (20 s) to threshold pressure (TP), Pmax, and ICI in Thy1-ChR2 mice. Photostimulation significantly reduced all of these parameters (No photostimulation vs. Photostimulation, mean ± s.e.m., TP: 12.5 ± 1.9 vs. 6.3 ± 1.4 mmHg, *p* = 0.0019, Pmax: 33.5 ± 7.3 vs. 29.4 ± 5.8 mmHg, *p* = 0.062, ICI: 6.7 ± 1.7 vs. 4.2 ± 1.2 min, *p* = 0.032, paired t-test, *p < 0.05, **p < 0.01, n = 5). (**f**–**h**) Effects of photostimulation in WT mice. There was no significant change in all the parameters (No photostimulation vs. Photostimulation, TP: 6.6 ± 1.8 vs. 5.6 ± 2.1 mmHg, *p* = 0.599, Pmax: 16.1 ± 2.3 vs. 16.1 ± 1.8 mmHg, *p* = 0.181, ICI: 4.3 ± 1.1 vs. 4.1 ± 0.8 min, *p* = 0.659, paired t-test). The blue and green dots represent the data for each individual in each condition, respectively, and the red dots represent the mean.

We found that only a brief period of photostimulation (2 s) could induce significant elevation of bladder pressure. The peak bladder pressure was dependent on the duration of photostimulation; longer stimulus duration induced larger peak bladder pressure (Fig. 2a and b, Supplementary Fig. S1b). The average increase in bladder pressure with photostimulation for 20 s was 8.5 ± 8.4 mmHg, which was consistent with that required to reach threshold pressure (TP) from the basal pressure observed in cystometrogram (CMG) of WT mice (~ 10 mmHg)^13^. Based on this condition, 20 s photostimulation was delivered every 1 min to examine the effect of optogenetic activation in ACC on the micturition parameters (Fig. 2c–h). In Thy1-ChR2 mice, photostimulation with constant intervals resulted in entrainment of the bladder contraction, which significantly shortened intercontraction interval (ICI), rather than the production of additional bladder contractions (Figs. 1c and 2e). Photostimulation also lowered TP and maximum bladder pressure (Pmax) (Fig. 2c and d). Only spontaneous bladder pressure changes were observed in WT mice regardless of photostimulation (Figs. 1d–f and 2f–h).

### Optogenetic stimulation of excitatory neurons in ACC induces bladder pressure increase

**Figure 3:**
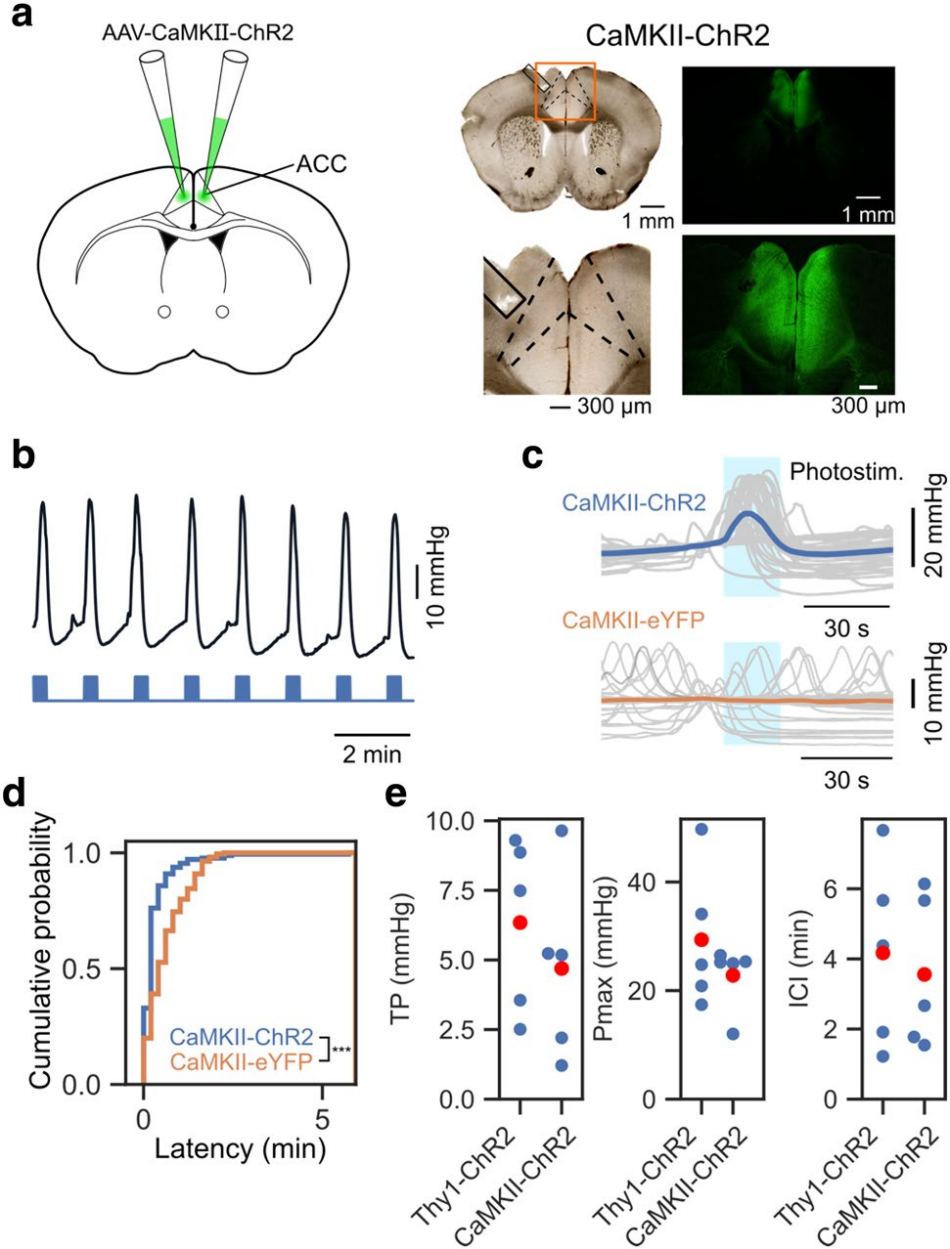
Optogenetic stimulation of excitatory neurons in ACC induces bladder pressure increase. (**a**) Left, AAV expressing ChR2 under CaMKII promotor was injected into ACC bilaterally. Right, Transparent and fluorescent micrograph showing localized expression of ChR2 in ACC. The boundaries of the ACC (dashed lines) and the tracks of optical fiber (solid lines) are marked. (**b**) A representative example of changes in bladder pressure during photostimulation of ACC. (**c**) Bladder pressure waveforms were aligned to the onset of photostimulation and overlaid. Upper, CaMKII-ChR2. Lower, CaMKII-eYFP. Gray lines indicate individual trials and colored lines indicate the mean. (**d**) Cumulative plots of the latency from the onset of photostimulation to the nearest peak of the bladder pressure changes. CaMKII-ChR2: 176 peaks from 6 mice, CaMKII-eYFP: 220 peaks from 4 mice. ***p < 0.001, Mann–Whitney U test. (**e**) Comparison of TP, Pmax, and ICI between Thy1-ChR2 and AAV-CaMKII-ChR2 injected mice. There were no significant differences in TP, Pmax, and ICI between Thy1-ChR2 mice and AAV-CaMKII-ChR2-injected wild-type mice. *p* values for TP, Pmax, and ICI were 0.55, 1.0, and 0.84, respectively. n = 5 mice, Mann–Whitney U test.

In Thy1-ChR2, almost all layer 5 pyramidal neurons in the cerebral cortex express ChR2. Thus, photostimulation might activate neurons in nearby cortical areas or passing fibers. To specifically express ChR2 only in excitatory neurons in ACC, we injected an adeno-associated virus (AAV) encoding ChR2, AAV-CaMKII-ChR2^14^, in ACC of WT mice (Fig. 3a and Supplementary Fig. S2a). Expression of ChR2 using this AAV is sufficient to induce action potentials upon photostimulation in CaMKII-expressing neurons in ACC (Supplementary Fig. S2b). Photostimulation to ACC in these mice induced robust elevation of bladder pressure (Fig. 3b–d). In contrast, photostimulation to ACC in control mice, which were injected with AAV-CaMKII-eYFP, failed to induce bladder pressure elevation (Fig. 3d). Furthermore, changes in TP, Pmax, and ICI upon photostimulation were not significantly different from those in Thy1-ChR2 mice (Thy1-ChR2 vs. AAV-CaMKII-ChR2; TP: 6.3 ± 3.1 vs. 4.7 ± 3.3 mmHg, *p* = 0.55, Pmax: 29.4 ± 13.0 vs. 22.8 ± 6.1 mmHg, *p* = 1.0; ICI: 252 ± 162 vs. 216 ± 132 s, *p* = 0.84; Mann Whitney U test, n = 5 mice each, Fig. 3e and Supplementary Table S1). These results indicate that selective activation of excitatory neurons in ACC reliably increased bladder pressure.

### Optogenetic stimulation of inhibitory neurons in ACC prolongs intercontraction intervals

**Figure 4:**
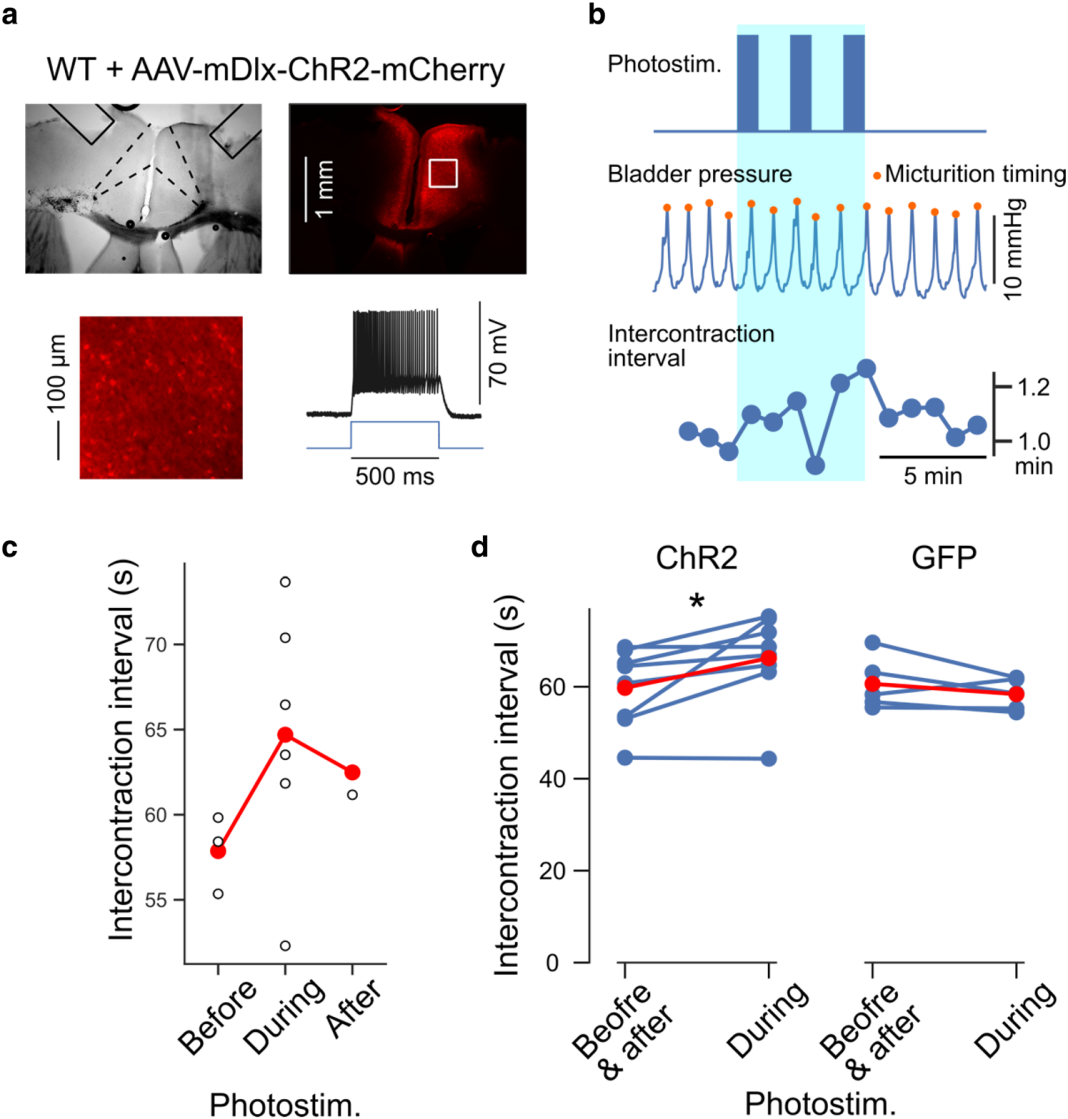
Optogenetic stimulation of GABAergic interneurons in ACC suppresses bladder pressure increase. (**a**) ChR2 was expressed in ACC of wild-type mice under the control of the mDlx enhancer element. Top, transmission and fluorescence micrograph showing localized expression of ChR2-mCherry in ACC. The boundaries of the ACC (dashed lines) and the tracks of optical fibers (solid lines) are marked. The image on the lower left represents an enlarged view of the mCherry fluorescence image framed by the white square in the upper right image. The lower right panel shows the action potentials in ChR2-mCherry expressing neurons induced by light irradiation. (**b**) A representative example of bladder pressure change during photostimulation to GABAergic interneurons in ACC. Top, the timing of photostimulation. Middle, bladder pressure. The orange markers indicate the micturition timing. Bottom, ICI. (**c**) ICI of bladder pressure was elongated during photostimulation. Data from (**b**). Open circle, each ICI. Red, mean. (**d**) Summarized data from ChR2- (8 mice) and GFP- (5 mice) expressing mice. ChR2, Before & after: 59.8 ± 3.0 s, During: 66.2 ± 3.5 s; GFP, Before & after: 60.6 ± 2.6 s, During: 58.4 ± 1.6 s. Blue, data from each mouse. Red, mean. *p < 0.05, Wilcoxon signed-rank test.

Activation of excitatory neurons in ACC triggered the increase in bladder pressure. Thus, we hypothesized that activating inhibitory neurons in ACC suppresses the increase in bladder pressure. To test this, we injected AAV-mDlx-ChR2-mCherry^15^ in ACC of wild-type mice to specifically express ChR2 in the GABAergic interneurons (Fig. 4a) and examined the effects of optogenetic stimulation to ACC on the bladder pressure. ACC neurons expressing ChR2 using this AAV-mDlx-ChR2-mCherry showed robust firing in response to light irradiation, whereas no action potential was induced by photostimulation in neurons expressing control AAV-mDlx-GFP (Supplementary Fig. S2c). The duration of optogenetically evoked action potentials of the mDlx-ChR2-expressing neurons was shorter than that of CaMKII-ChR2 expressing neurons (mDlx-ChR2: 1.1 ± 0.3 ms, CaMKII-ChR2: 3.7 ± 1.0 ms, Supplementary Fig. S2d), suggesting that the mDlx-ChR2-expressing neurons are fast-spiking GABAergic interneurons. In mice injected with AAV-mDlx-ChR2-mCherry, photostimulation significantly prolonged ICIs, whereas such prolongation of ICIs was not observed in control mice (Fig. 4b–d and Supplementary Fig. S3 and Supplementary Table S2). In addition, we injected AAV-DIO-ChR2 in ACC of PV-Cre mice^16^ to specifically express ChR2 in the parvalbumin (PV) -positive neurons (Supplementary Fig. S4a) and examined the effects of optogenetic stimulation to ACC on the bladder pressure. In these mice, photostimulation also significantly prolonged ICIs (Supplementary Fig. S4b*–*4d). Furthermore, simultaneous observation of bladder pressure and micturition reflex (Supplementary Fig. S5) showed that the timing of Pmax corresponded to that of voiding (Fig. 4b middle). Therefore, the bladder pressure increase and the micturition reflex was suppressed by the activity of local inhibitory interneurons in ACC.

### Pharmacological manipulation of neural activity in ACC bidirectionally modulates bladder pressure

**Figure 5:**
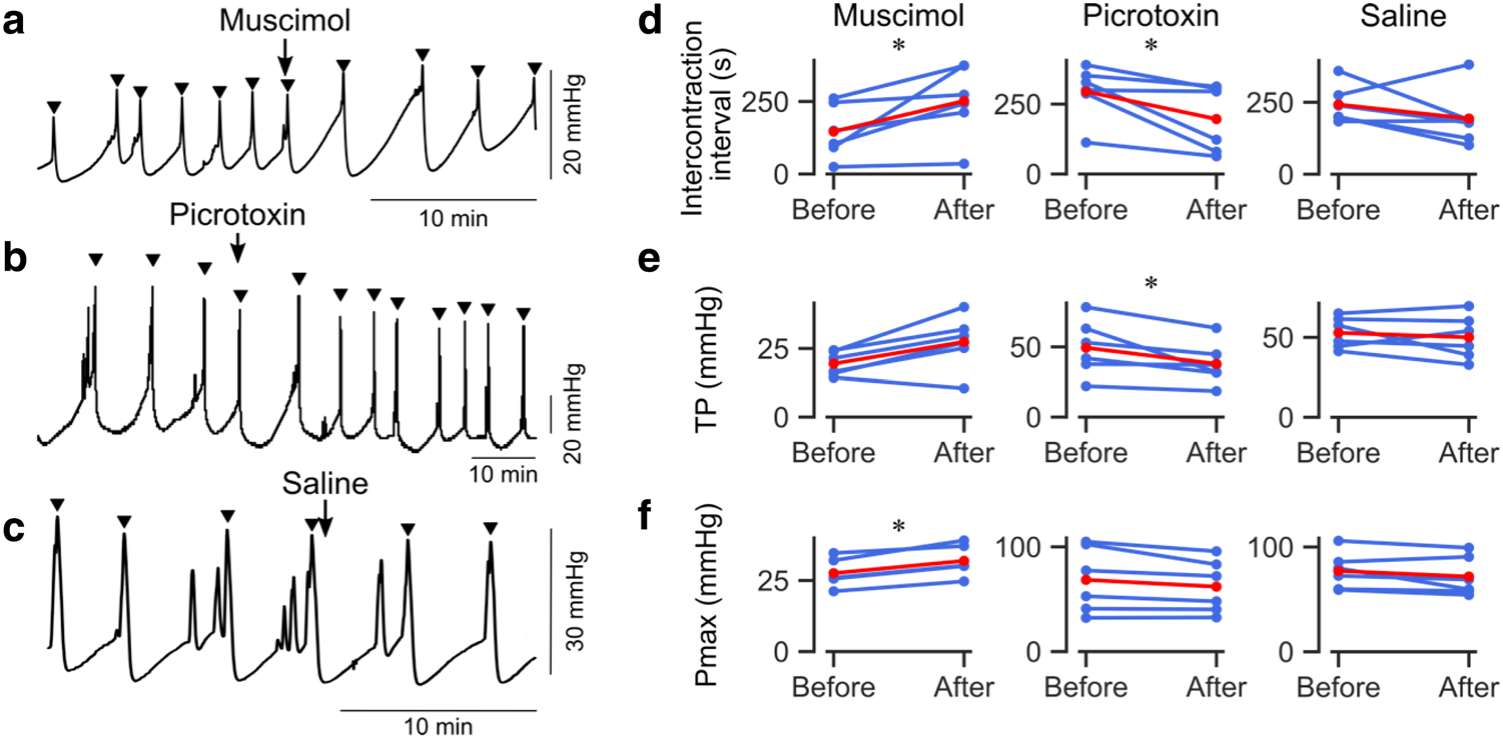
Effects of pharmacological manipulation of ACC on the bladder contraction. (**a**) A representative example of ICI changes by muscimol injection. Muscimol injection into ACC prolonged ICI. Filled triangles indicate the timing of Pmax. (**b**) A representative example of picrotoxin injection in ACC. Picrotoxin injection into bilateral ACC shortened ICI. (**c**) Saline injection into ACC did not change ICI. (**d**–**f**) Summary of changes in ICI, TP, and Pmax before and after the injections. Blue, data from each mouse. Red, mean. n = 6 each, *p < 0.05, Wilcoxon signed-rank test.

We further conducted pharmacological experiments to confirm the effect of ACC activity on the bladder pressure change. When muscimol, a potent and selective GABA_A_ receptor agonist, was injected into bilateral ACC, the ICI was significantly prolonged (Fig. 5a,d, and Supplementary Table S3). The result is consistent with the effect of optogenetic stimulation of GABAergic neurons. In contrast, the administration of picrotoxin, a GABA_A_ receptor antagonist, to ACC showed opposite effects, i.e., the ICI was shortened, and TP was lowered (Fig. 5b,d,e and Supplementary Table S3), consistent with the effect of optogenetic stimulation of excitatory neurons. All these results of optogenetic and pharmacological experiments suggest that an increase in the output from ACC promotes the increase in bladder pressure, and a decrease suppresses it.

### Optogenetic mapping of brain regions responsible for the induction of bladder pressure increase

**Figure 6:**
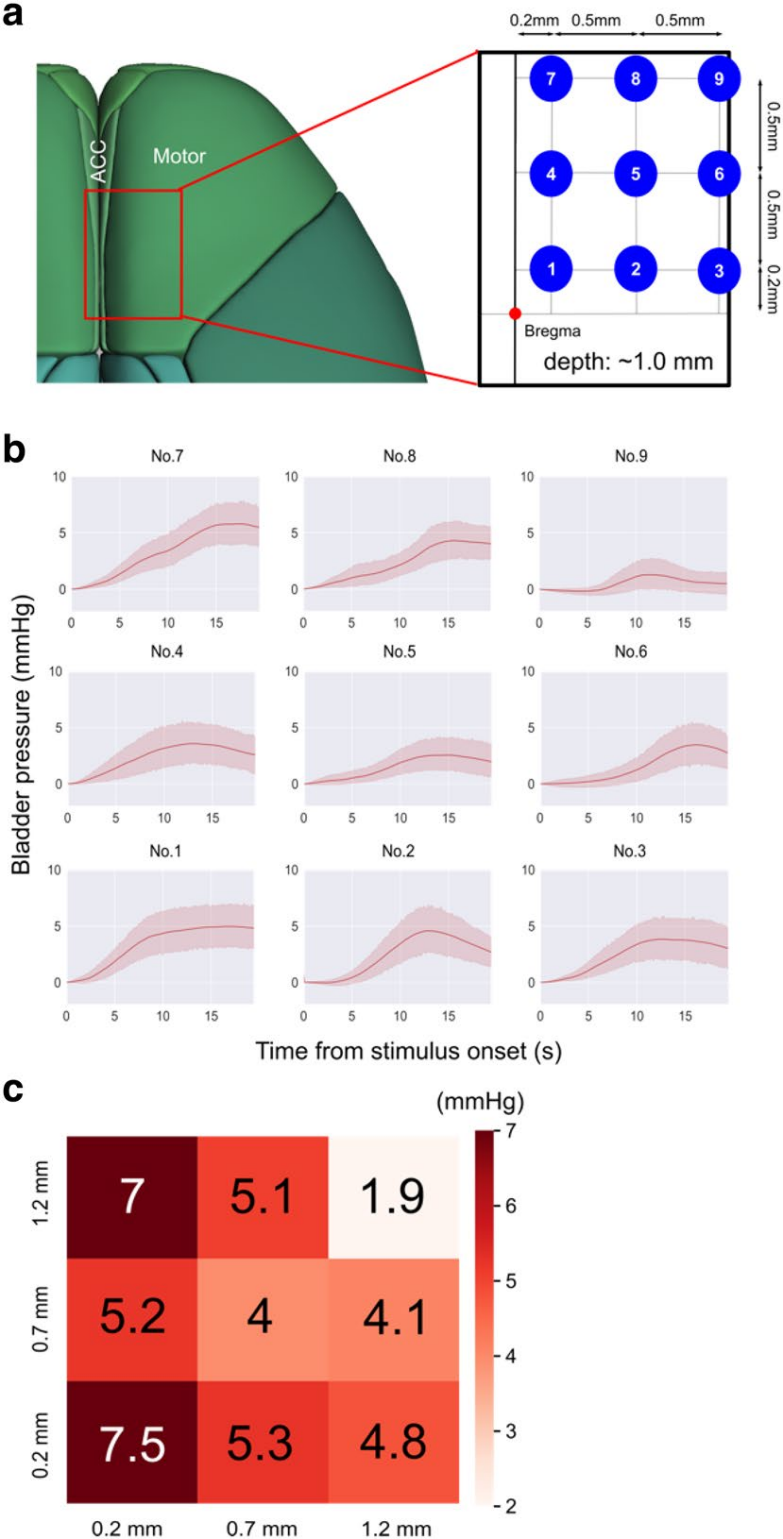
Optogenetic mapping of brain regions responsible for bladder pressure increase. (**a**) Location of photostimulation including ACC (medial, No.1, 4, 7) and the motor cortex (lateral, No. 3, 6, 9). The 3D brain atlas was created by Allen Brain Explorer^17^. (**b**) Bladder pressure waveforms in response to 20 s of photostimulation at each stimulation point. Thick lines and shadings are mean and 95% CI, respectively (n = 9 mice). (**c**) The heat map showing the average Pmax at each stimulation point. Photostimulation to ACC (left column: 1, 4, 7) showed a larger Pmax than the motor cortex (right column: 3, 6, 9).

Finally, to compare the contribution of ACC and nearby cortical areas to micturition, we performed optogenetic brain mapping experiments in Thy1-ChR2 mice (Fig. 6). A range of cortical regions, including ACC and the primary motor area anterior to bregma, was optogenetically stimulated while the bladder pressure was monitored (Fig. 6a and b). We stimulated neurons at the deep layer (~ 1.0 mm from the brain surface). The heat map of the average Pmax at each stimulation site showed that stimulation to ACC was more effective than to the motor cortex (Fig. 6c). Pmax was highest when stimulating ACC and decreased at the lateral part corresponding to the motor cortex. Therefore, the activity of ACC has a more significant impact on bladder pressure than the neighboring motor cortex.

## Discussion

In the present study, we examined the role of neural activity in ACC on the bladder pressure changes and micturition reflex. Using optogenetic stimulation, we investigated the role of excitatory and inhibitory neurons in ACC on bladder pressure. We found that activation of excitatory neurons in ACC induced a sharp increase in bladder pressure, whereas activation of inhibitory neurons prolonged the interval between bladder contractions. Moreover, pharmacological blockade and activation of GABA_A_ receptors in ACC promoted and suppressed bladder pressure, respectively. All these results indicate that the output from ACC controls the bladder pressure bidirectionally. Optogenetic mapping of cortical areas inducing bladder pressure increase revealed that stimulation of ACC is more effective than neighboring motor cortex to induce micturition. Thus, the results indicate that ACC is one of the powerful micturition centers.

Optogenetic stimulation of ACC in Thy1-ChR2 mice reliably induced bladder pressure increase. Thy1-ChR2 mice express ChR2 in a wide range of cortical layer 5 pyramidal cells. Therefore, it is possible that light stimulation using these mice may not be able to stimulate ACC specifically. Therefore, we conducted an experiment using mice injected with AAV-CaMKII-ChR2 in the ACC. As shown in Fig. 3, even in mice expressing ChR2 locally in the ACC, activation of excitatory neurons in the ACC by photostimulation caused an increase in bladder pressure. Furthermore, bladder contraction parameters (ICI, TP, and Pmax) were indistinguishable between Thy1-ChR2 mice and mice injected with AAV-CaMKII-ChR2 (Fig. 3e), suggesting that possible artifactual stimulation to neighboring cortical areas in Thy1-ChR2 was negligible.

Although previous human brain imaging studies suggested the involvement of ACC in LUT function, it was difficult to rule out the possibility that they detected neural activity that was not directly related to LUT function. This is because the results of these studies were not consistent. A study has reported that the activity of ACC was enhanced during the storage phase^8^. In contrast, other studies have reported seemingly contradictory results that ACC activity elevated during voiding phase^10^ or in patients with an overactive bladder^9^. Nevertheless, the prevailing hypothesis is that activation of ACC leads to suppression of micturition by promoting urethral sphincter contraction and suppressing detrusor contraction^18^. In fact, electrical stimulation to ACC in rodents has been shown to prolong the interval between bladder contractions^11^. Therefore, we expected that optogenetic stimulation of excitatory neurons and pharmacological inhibition of GABA_A_ receptors in ACC suppress bladder contraction. However, contrary to our expectation, all present findings indicate that ACC activity increases bladder pressure.

Optogenetic stimulation of inhibitory neurons and pharmacological activation of GABA_A_ receptors in ACC prolonged the interval between bladder contractions, indicating that increased inhibitory activity in ACC suppresses the sharp increase in bladder pressure. Thus, voiding and urine storage can be controlled by the activity of excitatory and inhibitory neurons in ACC, respectively. Inhibitory neurons in the cerebral cortex are classified into either parvalbumin (PV)-expressing cells, somatostatin (SST)-expressing cells, or ionotropic serotonin receptor (5HT3aR)-expressing cells^19^. In the ACC, the presence of PV-positive and SST-positive cells has been reported^20,21^. Among these GABAergic neurons, PV-positive cells are the most numerous^19,22^. Several reports showed that activating PV-positive cells in the barrel cortex using PV-Cre mice injected with AAV-DIO-ChR2 suppressed the responses of excitatory cells induced by sensory stimuli, demonstrating that activating PV-positive cells can inhibit excitatory neurons in that brain region^23^. In the present study, we utilized the mDlx enhancer^15^ to activate all types of GABAergic interneurons and showed that broadly activating GABAergic neurons in ACC suppresses micturition (Fig. 4). Furthermore, we also showed that activating PV-positive cells had a similar effect (Supplementary Fig. S4). The main objective of these experiments was to investigate the impact of suppressing excitatory activity in ACC. To gain insights into the role of each type of interneuron in the ACC for LUT functions, further studies will be necessary, which will involve manipulating the activity of specific types of interneurons within the ACC. Such research holds promise for advancing our understanding of the neural mechanisms underlying the control of LUT function.

The balance between excitatory and inhibitory activity in ACC may determine whether the micturition reflex is promoted or suppressed. Positive and negative bidirectional control might be an important characteristic of the regulation of bladder pressure by ACC, given that animals promote and suppress micturition depending on circumstances such as sensory cues and social context. ACC is known to be involved in processing pain, fear, and social decisions^24,25^. Thus, ACC may be a key regulator controlling downstream brainstem micturition centers to make a decision on whether to urinate or not. Further experiments in behaving mice will address this issue.

Optogenetic mapping experiments revealed that stimulation to ACC is more effective in inducing bladder pressure increase than the neighboring motor cortex. A recent report has shown that activating the caudal part of M1 induces the micturition reflex through PMC^7^. Our results have demonstrated that ACC has a similar role in LUT functions as the caudal M1, but the difference between ACC and M1 remains to be investigated. Furthermore, the downstream targets of ACC need to be determined. ACC is known to project to various brain regions, including micturition-related areas such as the frontal cortex, motor cortex, hypothalamus, PAG, and PMC^4^. Pathway-specific manipulation and recording of neural activity will clarify the role of each pathway in LUT functions.

In clinical practice, therapeutic procedures in patients with LUT dysfunction, such as indwelling urethral catheters or clean intermittent self-catheterization, are often performed to manage urological complications. However, these treatments may still cause further urological troubles during follow-up and affect the patient’s quality of life. Recently, several studies have shown that optogenetic stimulation to the bladder^26^, peripheral nerve^27,28^, and spinal cord^29^ can be utilized to control the LUT functions precisely, and these techniques are expected to be new therapeutic approaches. The present study opens the possibility of another method for controlling bladder pressure by manipulating brain activity and may contribute to the development of novel therapies for dysuria in the future.

## Materials and methods

All experiments and procedures were approved by the Animal Experiment Committee of University of Yamanashi (A1–20). All methods were carried out in accordance with the institutional and national guidelines and regulations, and are reported in accordance with ARRIVE guidelines.

### Mice

Thy1-ChR2-EYFP female mice^12^ were used for optogenetics experiments. Female C57BL/6J mice (C57BL/6JJmsSlc, Japan SLC) at 8–10 postnatal weeks were used in virus and drug injection and control experiments. PV-Cre female mice^16^ were used for optogenetic stimulation of PV neurons. The mice were housed in a room with a 12 h/12 h light–dark cycle at 24 °C, with 40–60% humidity. Food and water were provided ad libitum.

### Virus injection

Mice were anesthetized with isoflurane (2.5% for induction; 1.5–2% for maintenance). Mice were held by a stereotaxic apparatus (Narishige, Tokyo, Japan). The depth of anesthesia was monitored regularly by observing whisker movements and the pinch withdrawal reflex of the hindlimb. Body temperature was maintained at 36–37 °C using a heating pad. Lidocaine was topically applied to provide preemptive analgesia. Disinfect the skin surface with povidone-iodine and alcohol three times. A skin incision was made along the midline, exposing the skull over ACC. Small holes (0.2–0.5 mm in diameter, centered at 0.5 mm rostral to bregma, and 0.2 mm lateral from the midline on both sides of the hemisphere) were made for insertion of a glass pipette by using a dental drill. A pulled glass pipette was beveled to an outer diameter of 70 µm and loaded with a virus solution. Adeno-associated virus (100 nL; AAV2/1-CaMK2a-hChR2(H134R)-EYFP (addgene, Watertown, MA, #26969), AAV2/1-CamKII(1.3)-eYFP-WPRE-hGH(addgene # 105622), AAV2/1-EF1a-double floxed-hChR2(H134R)-EYFP-WPRE-HGHpA (addgene #20298), AAV2/1-mDlx-GFP-Fishell-1 (addgene #83900), and AAV2/1-mDlx-ChR2-mCherry-Fishell-3 (addgene # 83898); not diluted from original stock with ≥ 1 × 10^13^ µg/mL) was injected directly into bilateral ACC at a rate of 20 nL/min. The long axis of the pipette was angled at 20° from the vertical axis, and the tip of the pipette was advanced by 1.2 mm from the pia. After the injection, the pipette was kept in place for 5 min and removed. The skin incision was sutured, and additional lidocaine was applied to the suture wound. Anti-inflammatory and analgesic agents (dexamethasone and ketoprofen, 5 mg/kg each, i.p.) were administered to reduce stress and pain. After the surgery, mice were singly housed and allowed to recover for 14–21 days. We did not observe abnormal behavior after AAV injections.

### Cystostomy

Mice were anesthetized with urethane (1.5 g/kg, i.p.). The depth of the anesthesia was monitored regularly by observing whisker movements and the pinch withdrawal reflex of the hindlimb. The body temperature was kept at 36–37 °C using a heating pad. An abdominal midline incision was made, and the bladder was exposed. A pore was created in the bladder apex with a 20-gauge needle for bladder pressure measurements. After that, a polyethylene catheter (Clay-Adams PE50, Parsippany) was inserted gently through the bladder apex pore into the lumen. Purse string suture was performed to fix the catheter using 6–0 ethilon (ETHICON). The catheter was tunneled subcutaneously and anchored to the skin of the back with a 6–0 ethilon. Abdominal wounds were closed in layers.

### Cystometry

A catheter was connected to a perfusion apparatus (KD780220, Fisher Scientific) attached to a syringe pump. The perfusion rate was controlled at 10 μL/min. The bladder pressure induced by perfusing physiological saline was recorded by a pressure transducer placed in-line between a perfusion apparatus and the animal. Transducer outputs were digitized and recorded using a data acquisition system (Power Lab/8sp, AD Instruments) interfaced through a computer running vendor-supplied software (Power Lab Chart version 8.0, AD Instruments). The following cystometric parameters were measured: maximal voiding pressure (maximal bladder pressure during micturition), basal pressure, threshold pressure, and intercontraction interval (the intervals between each large amplitude spontaneous bladder contraction). Refer to Kadekawa et al. for the definition of urination terms^30^.

### Optogenetics

After cystostomy, the Thy1-ChR2-EYFP mouse was fixed with a stereotaxic frame (Narishige). Craniotomies 0.5 mm in diameter were made in both hemispheres (A + 0.5 mm, L ± 1.0 mm). Light probes (Cannula with Ø400 µm Core, 0.39 NA Multimode Fiber, CFML14L05, Thorlabs) were then inserted under stereotaxic assistance. The insertion angle was 45°, and the insertion depth was 1.8–2.0 mm. After insertion, light probes were fixed with dental cement. Cystometry experiments were performed 30–45 min after the craniotomy and probe implantation. After the intravesical bladder pressure stabilized, photostimulation (50 Hz, 0.01–60 s) was performed using an LED light source (Spectra X, Lumencor). The light power was 2.7 mW at the end of one of the two branched fibers (BFYL2LS01, Thorlabs). In brain mapping experiments, the range of craniotomy was the area enclosed by the right coronal suture, the midline, and the right inferior cerebral vein to avoid massive bleeding. We used light probes (Cannula with Ø105 µm Core, 0.22 NA Multimode Fiber, CFML21L05, Thorlabs). Coordinates of 9 insertion points were determined as follows: A: + 0.2, + 0.7, + 1.2 mm, L: + 0.2, + 0.7, + 1.2 mm. insert angle: 0°). The insertion depth was 1.0 mm from the surface. In experiments of locally expressing ChR2 in ACC, we performed cystometry and photostimulation after 3–4 weeks of virus injection. Photostimulation was delivered constantly during the measurement in all experiments except for the one shown in Supplementary Figure S1. To investigate whether photostimulation with fixed intervals affects bladder contraction, the ACC was randomly stimulated. The stimulation pulses at 50 Hz, with durations of either 0.5, 2, 5, or 20 s, and intervals of either 0.5, 1, or 2 min were generated using single-board microcontrollers (Arduino). Both the duration and the interval were chosen pseudorandomly from the values above.

### Measurement of micturition timing

The timing of micturition was measured simultaneously with the measurement of bladder pressure using the following method. First, to ensure the visibility of micturition and to prevent urine from spreading on the skin due to the fur, the hair around the vulva was removed with clippers and hair removal cream. Then, a catheter was surgically inserted into the bladder, and saline was infused to conduct cystometry. The mouse was then placed in the prone position, and the tail was lifted and held with a supporting rod to enable filming of the micturition process with a camera (Supplementary Fig. S5a). A urine receptacle made from a cut 2 cm piece of a plastic polyethylene dropper (1-4656-01, AS ONE Corporation) was placed under the vulva. To aspirate the urine discharged into the receptacle, a 200 µL micropipette tip was connected to a urethane tube, which was then attached to a vacuum pump (LV-125, Nitto Koki). The micropipette tip was then inserted into the urine receptacle. The discharge of urine and its suction were filmed using an industrial USB3.0 camera (DBK 33UX273, The Imaging Source). The image was captured every 0.1 s using an external trigger signal generated by a multifunction DAQ device (USB-6210, National Instruments). This external trigger signal for image capture was recorded simultaneously with the bladder pressure changes by the data acquisition system (Power Lab/8sp, AD Instruments). The timing of micturition was determined from the recorded camera image as follows. First, we compared two consecutive frames and calculated the difference in brightness for each pixel between them to create the frame-difference image (Supplementary Fig. S5b). We then manually selected a region of interest (ROI) in the frame-difference image, which included the area around the vulva, the urine receptacle, and the suction tip. We calculated the average pixel value within this ROI across all frames. To remove slow changes from the average pixel value of the ROI, we smoothed the waveform using the savgol filter and subtracted it from the original waveform. To standardize the data, we subtracted the minimum value and divided by the maximum value. Finally, we detected the timing of micturition by identifying the peaks using the find_peaks function of Python with a threshold (Median + 0.3 times the standard deviation) (Supplementary Fig. S5c).

### Drug administration

After the cystometry started, we monitored the bladder waveforms and waited until the waveform became stable. Subsequently, a glass pipette was inserted into bilateral ACC (position: A + 0.5 mm, L ± 1.0 mm, angle: 45°, insert depth: 2.2 mm). Picrotoxin (100 nL, 0.1 mg/mL, dissolved in saline) or muscimol (100–500 nL, 0.1 mg/mL, dissolved in saline) or saline were injected using a custom-made pressure injection device.

### Whole-cell patch-clamp recording in acute slice and optogenetic stimulation

Acute brain slices were prepared from the mouse brain after the bladder pressure measurements and the optogenetic experiments. The mice were transcardially perfused under deep anesthesia with the ice-cold artificial cerebrospinal fluid (ACSF; in mM): 124 NaCl, 2.5 KCl, 1.25 NaH_2_PO_4_, 26 NaHCO_3_, 2 CaCl_2_, 2 MgCl_2_, 10.1 glucose, saturated with 95% O_2_/5% CO_2_ gas. After decapitation, the brain was removed, and coronal slices of 300 μm thickness were made using a vibratome (VT1200S, Leica). The slices were prepared in the ice-cold ACSF. After incubation in a chamber at 35 °C for one hour, whole-cell recordings were conducted. ACSF bubbled with carbogen and heated to 30–35 °C using an inline solution heater (SH-27B, Warner Instruments) was perfused in a recording chamber installed under an upright microscope (BX51WI, Olympus), where the brain slice, including the ACC, was placed. Differential interference contrast (DIC) image of the slice was observed with an sCMOS camera (Orca-Flash 4.0 v3, Hamamatsu Photonics). Under the microscope, emission filters that could visualize the fluorescence of GFP, eYFP, or mCherry were used to search for cells expressing these proteins. Whole-cell recordings were conducted on cells expressing these fluorescent proteins. Borosilicate glass capillaries (GC150F-7.5, Harvard) were prepared using a micropipette puller (P-97, Sutter Instrument). The tip resistance was 4–8 MOhm. The electrode was filled with an intracellular solution for patching (in mM): 130 K-gluconate, 4 NaCl, 10 HEPES, 4 Mg-ATP, 0.3 Na-GTP, 7 dipotassium-phosphocreatine, pH adjusted to 7.0 with KOH (296 mOsm), and was approached to the cell to be recorded under positive pressure. Square wave pulses were applied to the tip of the electrode in voltage-clamp mode, and the resistance between the cell and the electrode was monitored using a patch-clamp amplifier (MultiClamp 700B, Molecular Devices, filtered at 10 kHz). After establishing the whole-cell recording, the membrane potential was measured in current-clamp mode. The recorded membrane potential data were digitized by a Multifunction I/O Device (NI USB-6343, National Instruments) at 20 kHz using custom data collection software written by LabView (National Instruments). An LED illumination system was used as the light source (X-Cite XYLIS, EXCELITAS Technologies). The cell was illuminated during whole-cell recording with light that has a wavelength of 480 ± 20 nm and an intensity of 2 μW for a duration of 500 ms.

### Histology

After physiological experiments, mice were deeply anesthetized with isoflurane or urethane and transcardially perfused with 4% paraformaldehyde (PFA) in phosphate-buffered saline (PBS). The brain was removed and post-fixed in 4% PFA at 4 °C for one day. Coronal sections of 50–100 µm thickness were obtained using a microslicer (DTK-1000N, Dosaka EM). For immunofluorescence, the sections were incubated at room temperature in PBS (5 min × 3) and in PBS containing 0.3% Triton X-100 (PBST) for 5 min. The sections were further incubated in the blocking solution (5% normal goat serum, 1% bovine serum albumin, and 0.3% Triton X-100 in PBS) for 60 min at room temperature. We then incubated the sections with a rabbit polyclonal antibody to parvalbumin (1:2000, Abcam) in the blocking solution at 4 °C overnight, washed with PBST (5 min × 3), and incubated with Alexa 568-conjugated anti-rabbit IgG (1:500, Abcam) for 2 h at room temperature. The sections were mounted on glass slides, and the fluorescence images were taken with an inverted fluorescence microscope (BZ-X800, Keyence).

### Statistics

Data are presented as mean ± s.e.m. unless otherwise stated. Statistical tests were performed by BellCurve for Excel (Social Survey Research Information Co., Ltd.) or the Python library of SciPy. All tests were two-tailed, and the significance level was set at *p* = 0.05.

### Supplementary Information

Supplementary Information.

## Data Availability

The datasets generated and analyzed during the current study are available from the corresponding author upon reasonable request.
