# Supplementary material for: Optogenetic stimulation of neurons in the anterior cingulate cortex induces changes in intravesical bladder pressure and the micturition reflex
Source: Sci Rep. 2024 Mar 16;14:6367. doi: 10.1038/s41598-024-56806-8 (PMC10944464; doi:10.1038/s41598-024-56806-8)
Supplement: Supplementary file 1 — Supplementary Information. [file 41598_2024_56806_MOESM1_ESM.docx]

Supplementary information

**Optogenetic stimulation of neurons in the anterior cingulate cortex induces changes in intravesical bladder pressure and the micturition reflex**

Takanori Mochizuki, Satoshi Manita, Hiroshi Shimura, Satoru Kira, Norifumi Sawada, Haruhiko Bito, Kenji Sakimura, George J Augustine, Takahiko Mitsui, Masayuki Takeda, Kazuo Kitamura


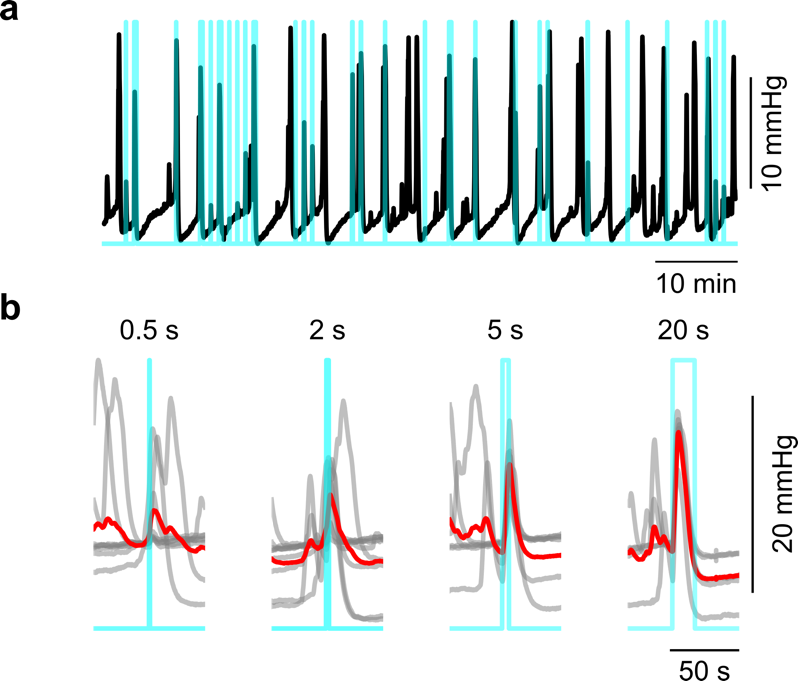


Supplementary Figure S1 | Bladder pressure changes observed with stimulation of the ACC at random timing

**a**. Bladder pressure changes when the ACC of Thy1-ChR2 mice was stimulated at random timings with variable stimulus durations. **b**. The bladder pressure changes around the stimulation timing were extracted, sorted by stimulus duration, and overlaid. The gray lines represent individual data, and the red lines represent the average of the data. The Pmax depended on the duration of the photostimulation.


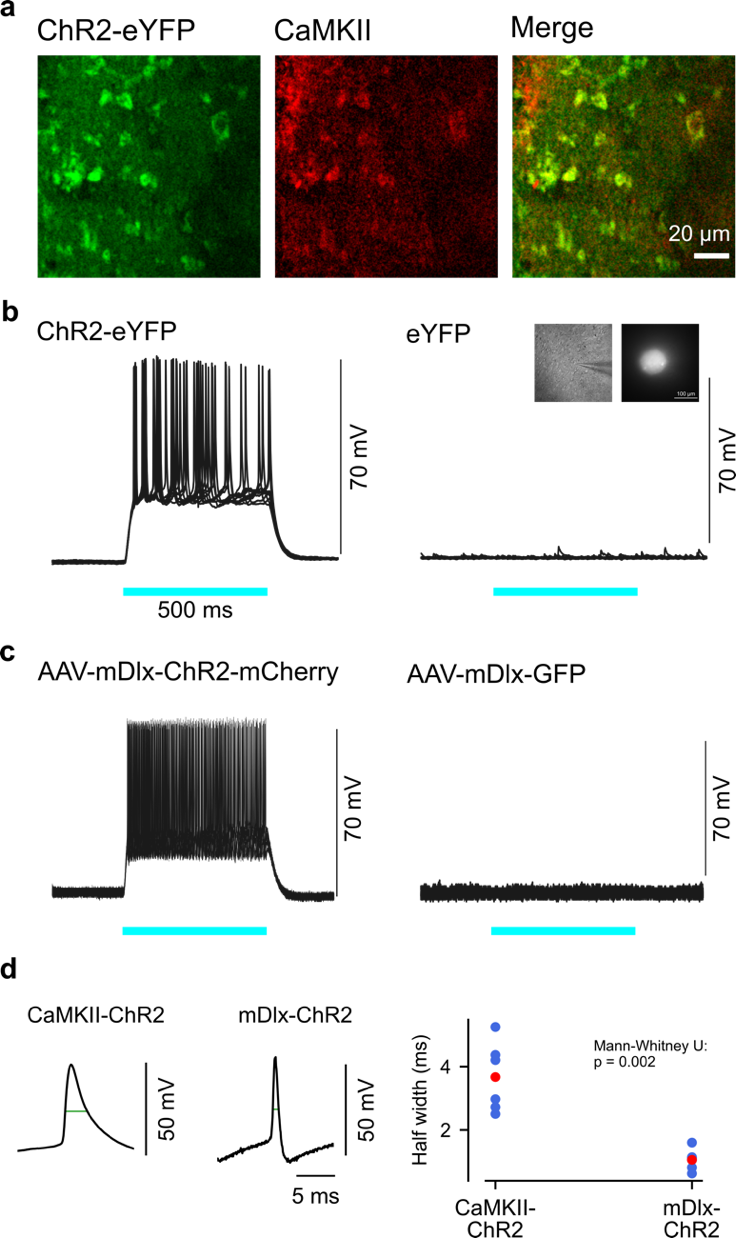


Supplementary Figure S2 | Optogenetic activation of excitatory and inhibitory neurons in ACC

**a**. A representative images of a section taken from a mouse injected with AAV-CaMKII-ChR2-eYFP immunostained with anti-CaMKII antibody. ChR2-eYFP-positive cells were CaMKII-positive. **b**. Blue light (2 µW) was irradiated to acute slices prepared from mice injected with AAV-CaMKII-ChR2-eYFP or AAV-CaMKII-eYFP while membrane potential of a ChR2- or eYFP-expressing neuron was recorded. The illumination of the light evoked action potentials in ChR2-eYFP-expressing neurons, but did not in eYFP-expressing neurons. **c**. The same experiments were performed in inhibitory neurons which expressed ChR2 or GFP with AAV-mDlx-ChR2-mCherry or AAV-mDlx-GFP, respectively. **d**. Left, representative optogenetically evoked action potential waveforms obtained from cells expressing ChR2 under the CaMKII promoter and cells expressing ChR2 under the mDlx enhancer. Green lines indicate half width. Right, summary of action potential half width. CaMKII: 3.7 ± 1.0 ms, mDlx: 1.1 ± 0.3 ms, n = 6 cells each.


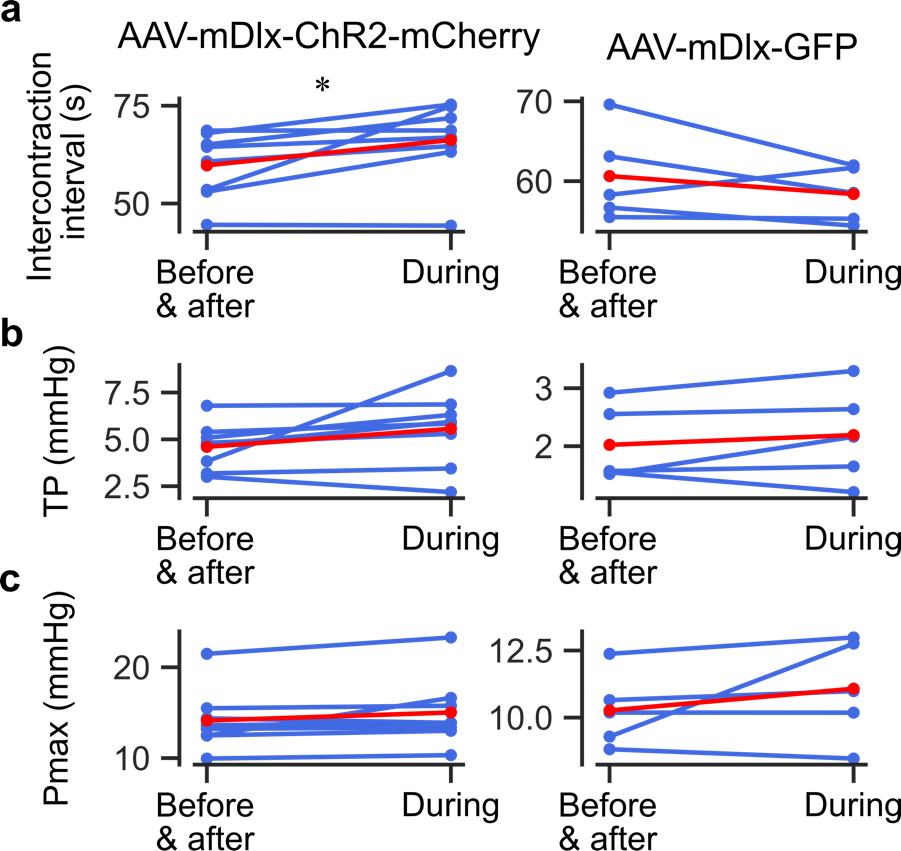


Supplementary Figure S3 | Effect of manipulation of inhibitory neurons in ACC on inter contraction interval, threshold pressure, and maximum pressure

**a–c**. Optogenetic stimulation of the ACC in wild-type mice injected with AAV-mDlx-ChR2-mCherry (left) or AAV-mDlx-GFP (right). The graphs show the intercontraction interval (**a**), threshold pressure (**b**), and maximum pressure (**c**) before and after or during photostimulation. The data before and after stimulation were combined for each mouse and averaged. Blue dots and lines represent data from individual mice, and red ones indicate the mean values. Data from 8 mice injected with AAV-mDlx-ChR2-mCherry and 5 mice with AAV-mDlx-GFP are shown. *p<0.05, Wilcoxon signed-rank test.


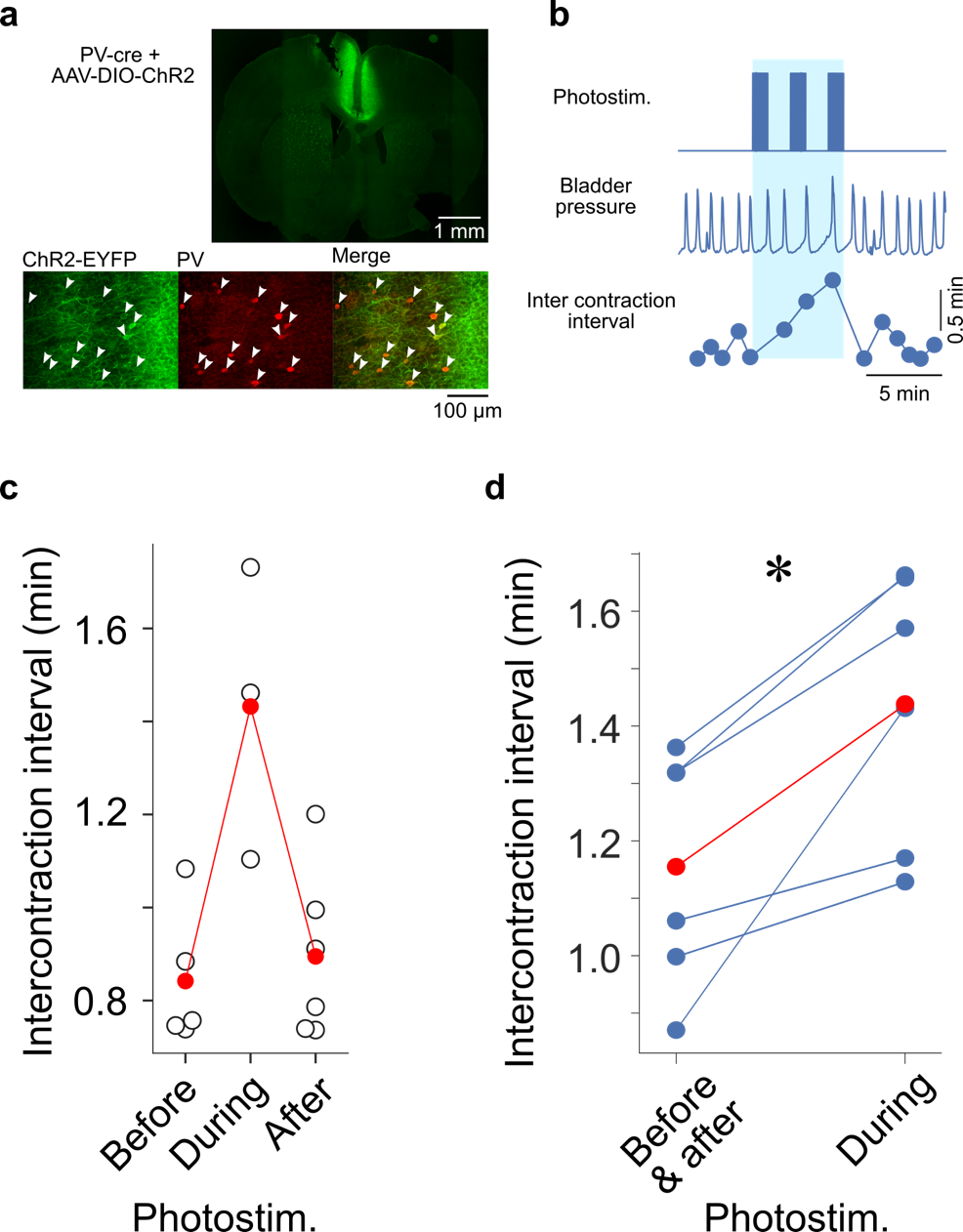


Supplementary Figure S4 | Optogenetic stimulation of PV-positive interneurons in ACC suppresses bladder pressure increase.

**a**, ChR2 was expressed in ACC of PV-Cre mice (upper). ChR2-EYFP-positive cells were PV-positive (lower). Arrowheads indicate cells in which EYFP and PV co-localize. **b**, A representative example of photostimulation of PV neurons in ACC. Top, the timing of photostimulation. Middle, bladder pressure. Bottom, ICI of the bladder pressure. **c**, ICI of bladder pressure was elongated during photostimulation. Data from **b**. Open circle, each ICI. Red, mean. **d**, Summarized data from 6 mice. Blue, data from each mouse. Red, mean. *p < 0.05, Wilcoxon signed-rank test.


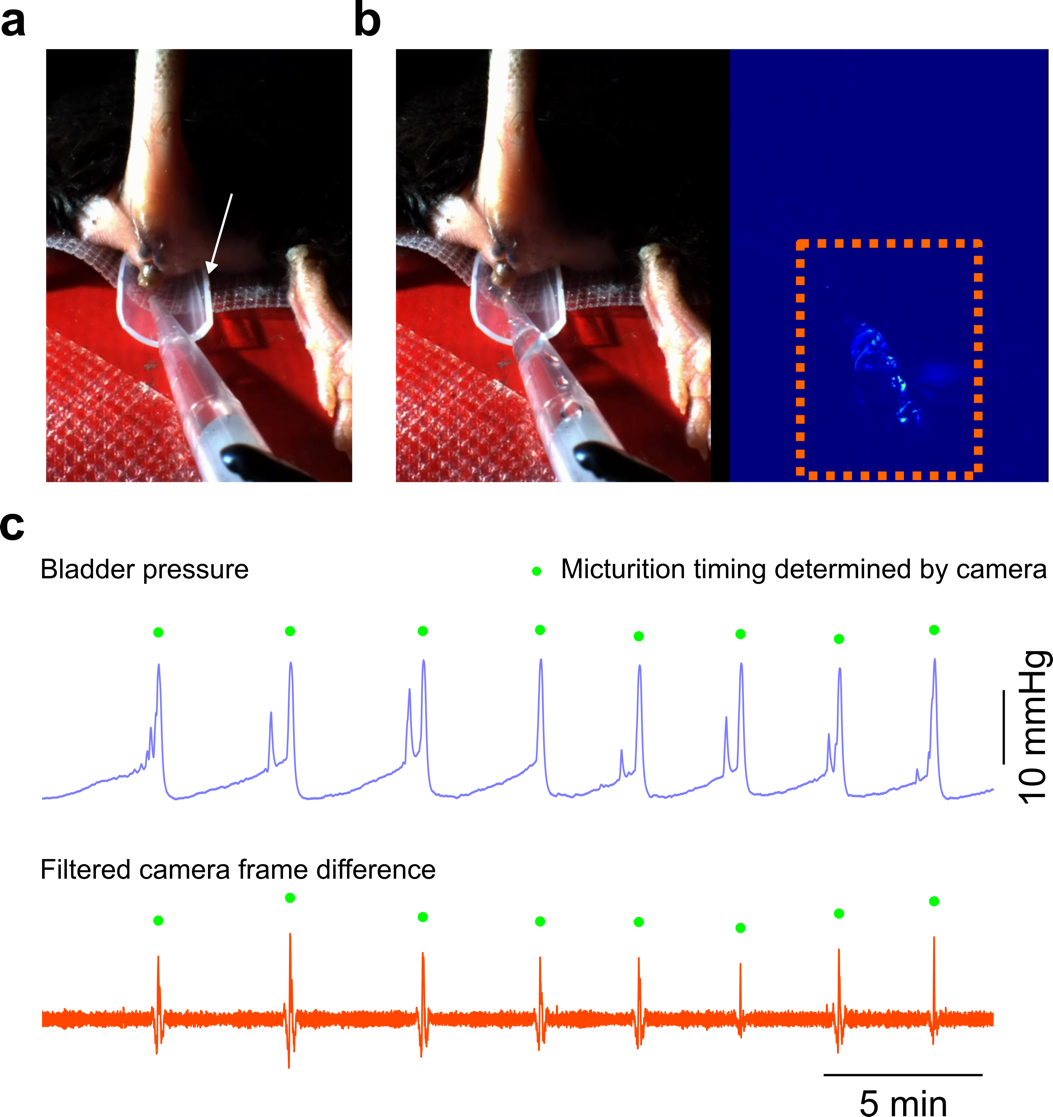


Supplementary Figure S5 | Measurement of micturition timing

**a**. Image showing the setup for measuring micturition. The arrow indicates a urine receptacle. A micropipette tip, which was connected to a vacuum pump, was used to collect the urine. **b**. The micturition was measured by calculating the difference in pixel values between two temporally consecutive frames (right). The average pixel value within the region of interest (ROI, orange square) across all frames was calculated. **c**. Upper, changes in bladder pressure. Lower, the average pixel value within the ROI. Green circles indicate the micturition timing measured from the peaks in the camera frame difference.

Supplementary Table S1 | Comparison of optogenetically induced bladder contraction parameters in Thy1-ChR2 mice and WT-mice injected with AAV-CaMKII-ChR2.

|  | Thy1-ChR2 | AAV-CaMKII-ChR2 | N | P value |
| --- | --- | --- | --- | --- |
| ICI (s) | 252 ± 162 | 216 ± 132 | 5, 5 | 0.84 |
| TP (mmHg) | 6.3 ± 3.1 | 4.7 ± 3.3 | 5, 5 | 0.55 |
| Pmax (mmHg) | 29.4 ± 13.0 | 22.8 ± 6.1 | 5, 5 | 1.0 |

Mann Whitney U test

Supplementary Table S2 | Effect of optogenetic activation of inhibitory neurons

| mDlx-ChR2 | Before & After stim. | During stim. | N | P value |
| --- | --- | --- | --- | --- |
| ICI (s) | 59.8 ± 3.0 | 66.2 ± 3.5 | 8 | 0.02* |
| TP (mmHg) | 4.6 ± 0.4 | 5.6 ± 0.7 | 8 | 0.07 |
| Pmax (mmHg) | 14.1 ± 1.2 | 15.0 ± 1.4 | 8 | 0.08 |

| mDlx-GFP | Before & After stim. | During stim. | N | P value |
| --- | --- | --- | --- | --- |
| ICI (s) | 60.6 ± 2.6 | 58.4 ± 1.6 | 5 | 0.31 |
| TP (mmHg) | 2.0 ± 0.3 | 2.2 ± 7.3 | 5 | 0.31 |
| Pmax (mmHg) | 10.3 ± 0.6 | 11.1 ± 0.8 | 5 | 0.44 |

*significance by Wilcoxon signed-rank test

Supplementary Table S3 | Summary of pharmacological experiments

| Muscimol | Before inj. | After inj. | N | P value |
| --- | --- | --- | --- | --- |
| ICI (s) | 146.5 ± 37.8 | 252.1 ± 51.2 | 6 | 0.03* |
| TP (mmHg) | 19.5 ± 1.8 | 27.4 ± 4.0 | 6 | 0.06 |
| Pmax (mmHg) | 27.6 ± 2.0 | 31.9 ± 2.2 | 6 | 0.03* |

| Picrotoxin | Before inj. | After inj. | N | P value |
| --- | --- | --- | --- | --- |
| ICI (s) | 295.0 ± 39.5 | 196.5 ± 49.0 | 6 | 0.03* |
| TP (mmHg) | 49.4 ± 8.1 | 37.9 ± 6.2 | 6 | 0.03* |
| Pmax (mmHg) | 68.5 ± 12.8 | 61.9 ± 10.4 | 6 | 0.06 |

| Saline | Before inj. | After inj. | N | P value |
| --- | --- | --- | --- | --- |
| ICI (s) | 242.2 ± 27.1 | 193.3 ± 40.3 | 6 | 0.44 |
| TP (mmHg) | 52.9 ± 4.0 | 50.0 ± 5.7 | 6 | 0.69 |
| Pmax (mmHg) | 77.1 ± 7.2 | 71.7 ± 7.7 | 6 | 0.16 |

*significance by Wilcoxon signed-rank test
